# Suicidal Behaviour and Related Risk Factors among School-Aged Youth in the Republic of Benin

**DOI:** 10.1371/journal.pone.0088233

**Published:** 2014-02-05

**Authors:** Jason R. Randall, David Doku, Michael L. Wilson, Karl Peltzer

**Affiliations:** 1 Centre for Injury Prevention and Community Safety (CIPCS), PeerCorps Trust Fund, Dar es Salaam, Tanzania; 2 Department of Community Health Sciences, University of Manitoba, Winnipeg, Canada; 3 Department of Population and Health, University of Cape Coast, Cape Coast, Ghana; 4 Unit of Adolescent Psychiatry, Turku University Hospital, Department of Adolescent Psychiatry, University of Turku, Turku, Finland; 5 Human Sciences Research Council, Pretoria, South Africa; 6 Department of Psychology, University of Limpopo, Mangkwang-E, South Africa; McGill University, Canada

## Abstract

**Introduction:**

Research on factors associated with suicidal ideation and suicide attempts has been conducted largely in developed countries. Research on West African countries in particular is lacking.

**Methods:**

Data were obtained from the Global School-based Health Survey conducted in Benin in 2009. This was a cross-sectional study of three grades, spanning Junior and Senior High, which sampled a total of 2,690 adolescents. Data on the occurrence of demographic, psycho-social and socio-environmental risk factors were tested using multinomial logistic regression for their association with suicidal ideation and suicide attempts.

**Results:**

The survey indicated that 23.2% had thought about suicide and 28.3% had made a suicide attempt in the previous year. Anxiety, loneliness, being bullied, alcohol misuse, illicit drug use, and lack of parental support were independently related to the ideation outcomes, suicidal ideation without planning and suicidal ideation with planning. Multinomial regression analysis, using one suicide attempt and multiple suicide attempts as outcomes, revealed that female sex, anxiety, loneliness, being physically attacked, and illicit drug use were associated these outcomes.

**Discussion:**

The prevalence of suicide attempts reported in the survey is relatively high. It is possible that there are cultural factors that could explain this finding. Our research indicates that many factors are related to the occurrence of suicidal ideation and suicide attempts among youth in Benin. Illicit drug use and violence in particular are associated with a high rate of suicide attempts in Benin. Measures to address these issues may reduce the risk of self-inflicted violence.

## Introduction

Suicide is of global public health importance. It is currently the 10th most common cause of mortality resulting in the loss of approximately one million lives every year [1], [2]. Among young people, suicide is estimated to contribute to 6% of all deaths worldwide [3]. Much of the current epidemiological evidence on the incidence and prevalence of suicidal ideation and suicidal behaviours is based on data from mainly high-income countries (HIC) [1]. This research has shown that psychosocial factors such as bullying, poverty, substance abuse and weak social relationships have been found to be related with suicidal thoughts and behaviours [4]–[6].

Comparatively, there exists little research on the occurrence of suicidal thoughts and behaviours in low-income countries (LIC) [1], [7]. Research on suicidal behaviours has been absent from several countries in sub-Saharan Africa (SSA), West Africa in particular. Some have suggested that there may be a hidden epidemic of suicide among the less developed countries including those in SSA where data is limited [8]. Previous research has estimated the one-year prevalence of suicide attempts at 12% in Nigerian adolescents [9]. This is higher than the one-year rates found for adolescents in western countries [10]–[12]. These studies estimated prevalences of 4.4% in Boston, USA, 4.9% in Nova Scotia, Canada and 6.3%–8.1% in nationally representative samples of American students [10]–[12]. The lifetime prevalence of self-harm by the age of 16/17 in the United Kingdom has been estimated at 18.8% [13]. In Norway the lifetime rate of suicide attempts for those 12–20 years old is 8.2%, with 2.7% having made an attempt in the last two years. A review of the literature on suicidal behaviour has found that both life-time attempt and last-year attempt prevalence are less than 10% in the majority of studies [14].

The true scope of the issue in West Africa is hidden by incomplete surveillance and socio-cultural issues surrounding suicide and its related stigma. For example, in some settings a suicide attempt is treated as a criminal offense further obscuring reporting [15], [16]. Very little research has been conducted on suicidal behaviours in Benin and the prevalence of suicide attempts among youth is unknown. One study in Benin found a lower prevalence of suicidal ideation, compared to western countries, among adult outpatients with depression [17].

Recently researchers have begun to utilize the Global School-based Health Survey (GSHS) to examine the occurrence of suicidal thoughts and behaviours [7], [18], [19]. Studies using the GSHS have examined the occurrence and correlates of suicidal ideation and behaviours in the Seychelles, Kenya, Botswana, Namibia, Zimbabwe, Tanzania, and Uganda [18], [20], [21]. These studies have found that 16%, and 11.2% of students had suicidal thoughts from the Seychelles, and Tanzania endorsed having suicidal ideation. This is comparable to the rates found in high income countries (HICs; 12.5%–16.7%) [10], [11], [22]. Other African countries, such as Kenya, Uganda, Botswana, and Zimbabwe, have rates that are noticeably higher than in HICs [20]. Further information is required to determine the burden of suicidal thoughts and behaviours among African youth, and in Benin in particular.

Risk factors such as psychological distress, exposure to bullying and violence, parental involvement, and alcohol and illicit drug abuse have been found to be associated with an increased risk of a suicide attempt in previous research [9], [14], [24]–[26]. A stress-diathesis model is a possible explanation for why these factors increase the risk of suicide [1], [27]. Since these factors often translate into increased stress they can lead individuals with the appropriate diathesis traits to turn to suicide as a means of escaping [1], [27]. Therefore any psycho-social or environmental factor measured by the GSHS could be a risk factor for suicidal behaviour in Benin.

Most of the research on risk factors for suicide attempts has been done in HIC countries and it is uncertain which risk factors are most important in Benin. However previous research has shown that mental disorders are involved in a smaller proportion of suicide deaths in LIC compared to HIC [28]. Therefore it is possible that other psycho-social and environmental factors may contribute to a larger portion of suicidal behaviours in Benin. Determining which of these factors are relevant can assist in determining interventions better suited for the youth of Benin. Psycho-social and environmental factors comprised a large portion of the questions asked in the GSHS performed in Benin. Therefore this survey provides an opportunity to examine the effect of these risk factors in Benin.

This study utilized data from the Republic of Benin contribution to the GSHS in 2009. Benin is a sub-Saharan country on the western coast of the continent currently classified by the World Bank as a low-income country [23]. The main objective of this study was to determine the prevalence of suicide attempts and investigate the association between demographic, psycho-social and socio-environmental risk factors and suicide attempts among school youth in Benin using Benin's version of the GSHS. The secondary objective of this study was to analyse the prevalence of suicidal ideation and its risk factors. This objective was included because many GSHS surveys only assessed ideation and not suicide attempts. Therefore including this outcome will allow some comparison to be made to the countries without suicide attempt data.

## Methods

### Ethics statement

Consistent with the GSHS study protocol [29], questionnaires were administered to all eligible participants in an anonymous, voluntary manner. Written permission had been obtained from each participating school and from all classroom teachers. Parental consent was also obtained. The study was approved by the Republic of Benin Ministry of Health.

### Sample

These data were collected in the Republic of Benin, a sub-Saharan African country located in West Africa. Data were obtained as part of the Global School-based Student Health Survey (GSHS). Developed collaboratively between the World Health Organisation (WHO) and the U.S. Centers for Disease Control (CDC), the GSHS has collected behavioural and health information from school-attending youth in 43 countries. The survey in Benin was conducted from November 7^th^ to December 24^th^ 2009 in 30 schools selected within twelve counties. The questionnaires were self-administered by students in class during school hours. The survey was anonymous and students were free to either participate or not participate. A two-stage cluster sample design was used to produce data representative of students in Grades “4eme”, “3eme”, and “seconde” in Benin. These grades span Junior and Senior High School in the French school system. At the first stage, schools were chosen with a selection probability proportional to their enrolment size. At the second stage, classrooms within each of the selected schools were randomly chosen. All students within selected classrooms were eligible to participate. The school response rate was 100% with 90% of the students choosing to participate. A total of 2 690 students participated in the Benin contribution to the GSHS. Full information on the procedures, methods and questionnaires that comprised data collection are described elsewhere [29]. The public dataset is also available through the WHO (http://www.who.int/chp/gshs/en/). Participants were 66.9% male. Almost half, 49.3%, of the sample was 16 years or older, which was the highest age option on the survey. Fifteen year-olds comprised 24.6%, and those aged 14, 13, and 12 and under comprised 14.8%, 7.0%, and 4.3% respectively.

### Measures and response coding

The questions used in the GSHS survey were jointly developed by the WHO and CDC. The survey contained one question regarding suicidal behaviour and two questions regarding suicidal ideation and planning. These questions were used to derive two categorical outcome variables with three levels in each, one variable for suicidal behaviour and one variable for suicidal ideation and planning. The questions used from the GSHS for the suicidal ideation and planning variable were: “*During the past 12 months, did you ever seriously consider attempting suicide?”* and *“During the past 12 months, did you make a plan about how you would attempt suicide?”*. Those who responded ‘*yes*’ to considering suicide but ‘*no*’ to planning were coded as suicidal ideation only (coded as 1) whereas those who responded ‘yes’ to both were coded as suicidal ideation and planning (coded as 2). Those who responded ‘no’ to both questions were coded as no ideation (coded as 0). For the suicidal behaviour outcome students were asked “*During the past 12 months, how many times did you actually attempt suicide?”*. Available responses were; “*0*”, “*1*”, “*2 or 3*”, “*4 or 5*”, and “*6 or more times*”. These data were recoded so that respondents were grouped into 3 categories; no attempts (coded as 0), 1 attempt (coded as 1) and multiple attempts (coded as 2).”

The independent variables extracted from the questionnaires included: sex, anxiety, loneliness, bullying, number of close friends, food insecurity, parental support, truancy, smoking, drinking, and substance use. The relevant questions for the independent variables and coding are indicated in **Table 1**.

**Table 1:** Independent variable derivation from survey data.

Survey Question	Coding	Variable
***How old are you?***	11–16 years (coded categorically)	Age
***What is your sex?***	(1) Male	Sex
	(0) Female	
***During the past 30 days, how often did***	(1) Most of the time/always	Food insecurity
***you go hungry because there was not***	(0) Never/rarely/sometimes	
***enough food in your home?***		
***During the past 12 months, how often***	(1) Most of the time/always	Anxiety
***have you been so worried about***	(0) Never/rarely/sometimes	
***something that you could not sleep at***		
***night?***		
***During the past 12 months, how often***	(1) Most of the time/always	Loneliness
***have you felt lonely?***	(0) Never/rarely/sometimes	
***How many close friends do you have?***	(0) 0 close friends	Close friends
	(1) 1 close friends	
	(2) 2 close friends	
	(3) 3+ close friends	
***During the past 30 days, on how many***	(0) 0–2 times	Truancy
***days did you miss classes or school***	(1) 3 to or more days	
***without permission?***		
***During the past 30 days, on how many***	(0) 0 times	Bullying
***days were you bullied?***	(1) 1 or more times	
***During the past 12 months, how many***	(0) 0 times	Attacked
***times were you physically attacked?***	(1) 1 or more times	
***During the past 12 months, how many***	(0) 0 times	In a fight
***times were you in a physical fight?***	(1) 1 or more times	
***During the last 30 days, on how many***	(0) 0 times	Smoking
***days did you smoke cigarettes?***	(1) 1 or more times	
***How old were you when you first used***	(0) I have never used drugs	Illicit drug use
***drugs?***	(1) Any other response	
***During your life, how many times did***	(0) 0 times	Alcohol misuse
***you drink so much alcohol that you***	(1) 1 or more times	
***were really drunk?***		
**Parental Support Index**		
**Created by adding the results of the coding below**		Parental support
***During the past 30 days, how often did***	(1) Most of the time/always	Parental homework checking
***you parents or guardians check to see***	(0) Never/rarely/sometimes	
***if your homework was done?***		
***During the past 30 days, how often did***	(1) Most of the time/always	Parental understanding
***your parents or guardians understand***	(0) Never/rarely/sometimes	
***your problems and worries?***		
***During the past 30 days, how often did***	(1) Most of the time/always	Parental knowledge of activity
***you parents or guardians really know***	(0) Never/rarely/sometimes	
***what you were doing with your free***		
***time?***		
***During the past 30 days, how often did***	(0) Most of the time/always	Parental intrusion of privacy
***your parents or guardians go through***	(1) Never/rarely/sometimes	
***your things without your approval?***		

### Statistical analysis

The distribution of students across the two outcome variables (suicidal ideation and planning, and suicide attempt) was tabulated and significant differences were determined for the risk factors of interest. Pearson's χ^2^ was used for categorical variables and ANOVA was used for continuous variables to determine variables which were significantly related to the two categorical outcomes. Multinomial logistic regression was used to examine the influence of all of the factors simultaneously. Multinomial regression was used due to the lack of a binary outcome variable precluding the usage of logistic regression. For the first multinomial logistic regression those without ideation were used as the baseline group and compared to those with ideation only, and those with both ideation and planning. For the second regression those with no reported suicide attempts were used as the baseline and compared to those with one attempt, and those with multiple attempts. Results of the regression analysis were reported using relative risk ratios (RRR) with 95% confidence intervals (CI). Multinomial regression was performed for the suicidal ideation groups and the suicide attempts groups separately. For the multinomial regression analysis, complete information was available for 2 115 participants (78.6%) of the sample. Due to the occurrence of missing responses for the questions about bullying, alcohol, and illicit drug use, multiple imputation was utilized to examine the possibility of bias. Analyses was done using Stata SE version 13.0 [30] for Windows.

## Results

### Suicidal ideation and planning

The risk factors among the three ideation groups are displayed in **Table 2**. 76.8% of the youth did not report any suicide ideation. The majority of the remaining students (17.5%) endorsed both suicide ideation and planning. The remaining 5.7% reported ideation with no planning. There were no significant relationships between age, sex, truancy, and number of close friends and suicidal ideation and suicide planning. Anxiety, loneliness, being bullied, being attacked, getting into fights, food insecurity, alcohol use, substance use, tobacco use, and parental support were all significantly associated with suicidal ideation and planning.

**Table 2:** Association of risk factors and suicide ideation among Benin adolescents.

Variable	Sample	No ideation	Ideation only	Ideation with plan	P-value
		%(n)**	%(n)**	%(n)**	
	100%	76.8%(2087)	5.7%(139)	17.5%(454)	
**Demographic**					
Age					0.0927
12 and under	4.3%	4.1%(67)	4.9%(6)	4.71%(18)	
13	7.0%	6.6%(109)	13.5%(16)	6.2%(22)	
14	14.9%	15.5%(271)	10.6%(14)	13.7%(53)	
15	24.7%	25.2%(483)	26.5%(33)	21.8%(91)	
16 and over	49.2%	48.6% (1150)	44.6%(69)	53.6%(269)	
Sex (male)	67.1%	67.0%(1359)	63.5%(83)	68.6%(296)	0.4261
**Psycho-social**					
Anxiety	17.7%	14.5%(312)	20.3%(32)	31.1%(147)	**<0.0001**
Loneliness	17.1%	14.5%(312)	17.5%(23)	28.3%(136)	**<0.0001**
**Socio-environmental**					
Bullied	13.7%	11.6%(222)	15.6%(19)	22.5%(101)	**<0.0001**
Attacked	35.8%	33.2%(674)	45.3%(62)	44.1%(195)	**0.0045**
In a fight	30.2%	28.4%(565)	29.6%(40)	38.3%(170)	**0.0043**
Truancy	29.1%	3.7%(70)	1.8%(8)	4.5%(31)	**0.3788**
Food insecurity	17.7%	16.5%(326)	24.6%(33)	21.0%(95)	**0.0103**
Alcohol misuse	17.0%	15.3%(314)	10.1%(13)	26.7%(116)	**<0.0001**
Substance use	4.2%	2.8%(51)	7.8%(9)	9.0%(36)	**<0.0001**
Tobacco use	3.6%	2.7%(54)	6.1%(8)	6.7%(31)	**0.0004**
Parental support*					**0.0129**
0 (low support)	1.5%	1.1%(22)	2.7%(3)	3.0%(13)	
1	30.0%	28.5%(620)	28.9%(40)	37.1%(176)	
2	27.4%	27.7%(561)	27.0%(40)	26.2%(118)	
3	24.0%	24.4%(484)	31.8%(42)	20.1%(88)	
4 (high support)	17.0%	18.4%(368)	9.7%(13)	13.6%(54)	
Close friends					0.1051
0	12.0%	11.99%(234)	18.34%(25)	9.75%(46)	
1	23.3%	23.11%(481)	26.68%(38)	22.74%(100)	
2	17.8%	17.05%(364)	16.46%(22)	21.44%(99)	
3 or more	47.0%	47.85%(1000)	47.85%(54)	46.07%(208)	

* Parental support tested as a continuous variable ** weighted % and unweighted n.

The results of the multinomial logistic regression are presented in **Table 3**. After adjusting for all variables the only three factors which remained statistically significant in the regression model for ideation only were; anxiety (RRR = 2.16; 95%CI: 1.31–3.56), substance use (RRR = 3.06 95%CI: 1.12–8.34), and having 0 close friends (compared to 3+ close friends; RRR = 1.98; 95%CI: 1.06–3.68). For those with both ideation and planning, six factors were found to be significant. Anxiety (RRR = 2.34; 95%CI: 1.63–3.37), loneliness (RRR = 1.86; 95%CI: 1.30–2.67), being bullied (RRR = 1.47; 95%CI: 1.00–2.15), alcohol misuse (RRR = 1.52; 95%CI: 1.02–2.27), substance use (RRR = 1.69; 95%CI: 1.05–2.72), and parental support (RRR = 0.88; 95%CI: 0.80–0.96) were significantly associated with suicidal ideation and planning in the regression model.

**Table 3:** Multivariable multinomial logistic regression for association with ideation.

	Ideation only		Ideation w/plan	
Variable	RRR* (95%CI)	p-value	RRR* (95%CI)	p-value
Age				
12 and under	–	–	–	–
13	1.25 (0.71–5.19)	0.682	0.99 (0.28–3.48)	0.982
14	0.69 (0.30–1.85)	0.243	0.70 (0.27–1.81)	0.435
15	0.98 (0.46–2.07)	0.948	0.86 (0.28–2.60)	0.775
16	0.78 (0.35–1.74)	0.520	1.05 (0.36–3.00)	0.930
Sex (male)	0.85 (0.56–1.28)	0.412	1.06 (0.91–1.25)	0.428
**Psycho-social**				
Anxiety	**2.16 (1.31**–**3.56)**	**0.005**	**2.34 (1.63**–**3.37)**	**<0.001**
Loneliness	1.07 (0.60–1.92)	0.806	**1.86 (1.30**–**2.67)**	**0.002**
**Socio-environmental**				
Bullied	1.18 (0.63–2.20)	0.584	**1.47 (1.00**–**2.15)**	**0.048**
Attacked	1.71 (0.96–3.04)	0.066	1.23 (0.95–1.58)	0.107
In a fight	0.86 (0.60–1.23)	0.377	1.23 (0.84–1.78)	0.262
Food insecurity	1.46 (0.72–2.97)	0.269	1.11 (0.83–1.50)	0.455
Alcohol misuse	0.57 (0.31–1.05)	0.069	**1.52 (1.02**–**2.27)**	**0.043**
Substance use	**3.06 (1.12**–**8.34)**	**0.031**	**1.69 (1.05**–**2.72)**	**0.032**
Tobacco use	0.98 (0.37–2.61)	0.963	1.24 (0.58–2.63)	0.560
Parental support**	0.84 (0.68–1.04)	0.104	**0.88 (0.80**–**0.96)**	**0.008**
Truancy	0.31 (0.04–2.64)	0.261	0.81 (0.42–1.55)	0.491
Close friends				
3+	–	–	–	–
2	1.02 (0.53–1.93)	0.958	1.02 (0.71–1.46)	0.927
1	1.42 (0.82–2.45)	0.191	1.05 (0.71–1.53)	0.809
0	**1.98 (1.06**–**3.68)**	**0.034**	0.92 (0.51–1.68)	0.783

Unweighted n = 2115: Ideation only n = 106, Ideation and plan n = 366.

* RRRs adjusted for all factors which appear in table.

** Parental support is analyzed as a continuous variable.

### Suicide attempts

The students were also divided into three groups based on whether they indicated they had attempted suicide during the previous year. The three groups were no attempts, one attempt, and two or more attempts. **Table 4** shows the distribution of students in these three groups across the risk and protective factors. Seventy-two per cent of students did not have any suicide attempts. Within the previous year 28.3% endorsed making an attempt, 14.8% indicated one suicide attempt and 13.5% indicated that they had made multiple suicide attempts. All of the risk factors were individually associated with the occurrence of suicide attempts among the students except age and sex.

**Table 4:** Association of risk factors and suicide attempts.

Variable	No attempts	1 attempt	2+ attempts	P-value
	%(n)**	%(n)**	%(n)**	
	71.7%(1960)	14.8%(369)	13.5%(351)	
Age				0.1335
12 and under	3.8%(58)	5.8%(18)	5.2%(16)	
13	7.0%(107)	7.3%(23)	6.2%(17)	
14	14.9%(246)	18.2%(59)	10.7%(32)	
15	24.4%(437)	24.7%(85)	26.2%(85)	
16	49.8%(1106)	44.1%(182)	51.7%(200)	
Sex (male)	66.9%(1271)	66.5%(235)	67.5%(227)	0.9436
**Psycho-social**				
Anxiety	13.6%(286)	23.9%(89)	33.6%(118)	**<0.0001**
Loneliness	14.3%(289)	17.6%(71)	31.1%(110)	**<0.0001**
**Socio-environmental**				
Bullied	11.0%(201)	14.04%(49)	28.2%(93)	**<0.0001**
Attacked	29.7%(565)	49.2%(182)	53.9%(183)	**<0.0001**
In a fight	25.2%(477)	41.4%(148)	44.8%(150)	**<0.0001**
Food insecurity	16.3%(307)	20.2%(71)	22.4%(74)	**0.0020**
Alcohol misuse	14.4%(281)	18.7%(65)	28.6%(95)	**0.0002**
Substance use	1.9%(35)	7.6%(24)	13.5%(38)	**<0.0001**
Tobacco use	2.7%(50)	4.0%(14)	8.4%(29)	**0.0001**
Parental support*				**0.0001**
0 (low support)	1.3%(22)	2.6%(9)	2.6%(9)	
1	28.6%(588)	31.1%(122)	34.9%(123)	
2	26.9%(517)	29.9%(105)	27.6%(98)	
3	24.5%(456)	22.6%(79)	23.2%(80)	
4 (high support)	18.7%(349)	13.8%(49)	11.7%(37)	
Truancy	2.8%(49)	3.2%(12)	9.4%(33)	**0.0001**
Close friends				**0.0078**
0	13.2%(238)	9.2%(36)	8.3%(30)	
1	22.8%(446)	29.5%(109)	19.1%(65)	
2	16.4%(329)	21.7%(78)	21.4%(78)	
3 or more	47.7%(940)	39.6%(144)	51.2%(177)	

* Parental support tested as a continuous variable ** weighted % and unweighted n.

The results of the multinomial logistic regression for association with suicide attempts are shown in **Table 5** **.** Three factors were significantly associated with one suicide attempt in the last year. These variables were anxiety (RRR = 2.05; 95%CI: 1.37–3.07), being attacked (RRR = 2.04; 95%CI: 1.61–2.59), and sex (males; RRR = 0.74; 95%CI: 0.60–0.90). For those with multiple attempts, four of the variables were significant; anxiety (RRR = 2.43; 95%CI: 1.60–3.71), loneliness (RRR = 2.06; 95%CI: 1.44–2.97), substance use (RRR = 5.73; 95%CI: 2.99–11.0), and being attacked (RRR = 2.09; 95%CI: 1.40–3.11).

**Table 5:** Multivariable multinomial logistic regression for association with suicide attempts.

	1 suicide attempt		2+ suicide attempts	
Variable	RRR (95%CI)*	p-value	RRR (95%CI)*	p-value
Age				
12 and under	–	–	–	–
13	0.77 (0.33–1.76)	0.503	1.37 (0.26–7.23)	0.692
14	0.92 (0.40–2.13)	0.832	1.07 (0.32–3.63)	0.907
15	0.72 (0.35–1.46)	0.337	1.73 (0.45–6.66)	0.397
16	0.60 (0.26–1.41)	0.223	1.26 0.25–6.25)	0.763
Sex (male)	**0.74 (0.60**–**0.90)**	**0.005**	0.80 (0.55–1.15)	0.208
**Psycho-social**				
Anxiety	**2.05 (1.37**–**3.07)**	**0.002**	**2.43 (1.60**–**3.71)**	**<0.001**
Loneliness	0.85 (0.49–1.47)	0.541	**2.06 (1.44**–**2.97)**	**0.001**
**Socio-environmental**				
Bullied	0.73 (0.41–1.29)	0.260	1.61 (0.96–2.70)	0.070
Attacked	**2.04 (1.61**–**2.59)**	**<0.001**	**2.09 (1.40**–**3.11)**	**<0.001**
In a fight	1.45 (0.99–2.14)	0.057	1.48 (0.93–2.36)	0.091
Food insecurity	1.20 (0.92–1.55)	0.165	1.05 (0.73–1.51)	0.786
Alcohol misuse	1.11 (0.65–1.90)	0.672	1.62 (0.97–2.70)	0.063
Substance use	2.70 (0.93–7.85)	0.066	**5.73 (2.99**–**11.0)**	**<0.001**
Tobacco use	0.64 (0.31–1.31)	0.200	0.89 (0.39–2.03)	0.766
Parental support**	0.92 (0.78–1.09)	0.295	1.00 (0.73–1.51)	0.953
Truancy	0.79 (0.24–2.54)	0.669	1.70 (0.61–4.74)	0.290
Close friends				
3+	–	–	–	–
2	1.54 (0.93–2.54)	0.091	1.23 (0.80–1.92)	0.322
1	1.60 (0.98–2.60)	0.058	0.80 (0.42–1.53)	0.479
0	0.83 (0.47–1.45)	0.485	0.56 (0.31–1.01)	0.055

Unweighted n = 2117: 1 attempt n = 267, 2+ attempts n = 246.

* RRRs s adjusted for all factors which appear in table.

** Parental support is analyzed as a continuous variable.

### Multiple imputation analysis

The results of the multiple imputation analysis suggested that the analysis for factors associated with suicidal ideation was potentially biased by missing values. All three previously significant variables were no longer significantly associated with ideation: anxiety (RRR = 1.52; p = 0.066), having 0 close friends (RRR = 1.78; p = 0.062), and illicit drug use (RRR = 1.76; p = 0.221). Two variables became significant after imputation: being attacked (RRR = 1.76; p = 0.029), and alcohol misuse (RRR = 0.46; p = 0.026). For suicidal ideation and planning, alcohol misuse was no longer significant (RRR = 1.33; p = 0.139). This was the only difference for that outcome. Most of these variables had p-values that were already close to 0.05; therefore it is unsurprising that they shifted into or out of significance. However this was not true for anxiety and illicit drug use. These both experienced a significant reduction in their estimated RRRs and significant changes in their p-values.

The results of the regression for suicide attempts did not suggest serious bias. Several additional variables became significant, likely due to the increased power obtained by including more cases. For one suicide attempt, these variables were being in a fight (RRR = 1.54; p = 0.021), using illicit drugs (RRR = 3.08; p = 0.002), and having 1 or 2 friends (compared to 3+ friends; RRR = 1.67; p = 0.012, RRR = 1.64; p = 0.027). One variable ceased to be significant for the 1 attempt outcome: female gender was no longer predictive of a single suicide attempt (RRR = 0.88; p = 0.111). For the multiple attempt outcome the additional significant variables were bullying (RRR = 1.67; p = 0.039), being in a fight (RRR = 1.48; p = 0.036), and having no close friends (RRR = 0.57; p = 0.007).

## Discussion

The present study investigated the prevalence and risk factors for suicidal ideation and suicide attempts in the West African country of Benin. Overall the prevalence of suicidal ideation in Benin is comparable to the rates of ideation and planning found in other studies in SSA [18], [20]. The rate of suicide attempts during the one-year period of recall was high compared to the prevalence found in other low and middle income countries, such as Nigeria, Thailand, Vietnam, China and the Philippines. [9], [31], [32]. Suicide attempt prevalence was also higher in Benin compared to HICs such as Canada and the US, where estimated rates are between 4.4% and 9% [10], [14], [25], [26], [33]–[38].

### Suicidal ideation and planning

Most previous research on suicidal ideation has not made a distinction between ideation with or without planning. Therefore this discussion will focus on suicidal ideation without making a distinction based on the occurrence of planning. This sample of adolescents had a one-year prevalence of suicidal ideation of 23.2% which is comparable to those found in other SSA countries such as Uganda (19.6%), Botswana (23.1%), Kenya (27.9%) and Zimbabwe (25.5%) [19], [20]. It was higher than rates found in the Seychelles (16%), and Tanzania (11.2%). The rate of suicidal ideation was also higher than the rates found in the GSHS in the Eastern Asian countries of Thailand, China, Taiwan, Vietnam, and the Philippines [31], [49], [50]. The prevalence of suicidal ideation was also higher in Benin compared to HIC settings (12.5%–16.7%) [10], [12], [35], [36].

The occurrence of anxiety, loneliness, being bullied, alcohol misuse, illicit drug use, and low parental support were associated with increased occurrence of suicidal ideation and planning. Anxiety, loneliness, and low parental support were associated with increased suicidal ideation and planning in the GSHS from the Seychelles as well [18]. This study found no evidence for an association between tobacco use and suicidal ideation. Other research has found no association with tobacco use [51]. However, a multinational study using GSHS data indicated that tobacco use was related to suicidal ideation among girls in Africa specifically [52]. No significant differences by sex were observed in this sample of adolescents which conflicts with other research in the region [18], [20], [21].

### Suicide attempts

It is unclear why the rate of suicide attempts is so high among the youth of Benin. A number of context specific concerns might explain the higher rate of suicidal behaviours in Benin. The survey was conducted in 2009, which was a year of abnormal hardship for Benin. The global economic crisis was at its peak while simultaneously the country experienced significant flooding [39]. Also, the level of participation in a formal economy is lower in Benin compared with other countries in the region that have been examined using the GSHS. Previous research has indicated that SES and cultural factors significantly affect the occurrence of suicide [40], [41]. Therefore the low level of prosperity in Benin, compared to other developing countries, may partially explain the data variation.

Previous studies have shown that suicide attempts among young people are associated with critical life-course experiences which cause stress and shame in the family [42], [43]. In LIC settings, law enforcement is weak, and there are few institutionalised social safety nets for vulnerable youth other than the family during critical life circumstances such as the loss the family's main income-earner. Moreover, in most LIC settings, events such as sexual and physical abuses are shrouded in secrecy. With these prevailing circumstances young people may resort to suicidal behaviours as a way of dealing with adverse life circumstances. There has been some research into the high rate of suicide attempts among Latina youth in the US that has discussed possible cultural factors and how they relate to suicidal behaviour [41]. In particular Zayas et al. suggest that many suicide attempts in Latinas may be due to a cultural idiom of distress [43]. Essentially suicidal behaviour may be a culturally bound means of expressing distress. Benin may also have a similar idiom of distress that causes youth there to engage in suicidal behaviour more readily as a response to, and means of conveying, distress. It could also explain why the rate of attempts is higher than the rate of ideation. In American Latinas the idiom of distress resulted in impulsive suicide attempts. These acts were described as suicidal but were not preceded by a clear occurrence of ideation [41]. However, for Benin, the specific aspects of SES, culture and their variable effect on the occurrence of suicide is uncertain due to a lack of research in the country.

Consistent with previous findings, sex, anxiety, loneliness, interpersonal violence, and illicit drug use were all associated with increased suicide attempts [44]–[47]. Illicit drug use was the biggest risk factor for suicide attempts in this sample. However due to the frequent occurrence of violence among the respondents, 48.3% having been attacked or being involved in a fight, violence was related to a larger portion of the suicide attempts in this sample. It is possible that youth violence, and potentially gang-related violence, is elevated in Benin. This may explain why those with more friends were more likely to engage in suicidal behaviours. Other research has found a protective effect for those with multiple close friends [45]. If group violence is common then having many close friends may indicate membership in a group prone to violence. This may result in an increased density of factors which are enablers of suicidal behaviours. This may also explain the weak sex discrepancy in suicide attempts if males in Benin were more likely to be involved in gangs, as they are in western countries [48]. This exposure may counteract the more commonly reported situation of females having higher rates of suicidal behaviours. However we were unable to locate any clear quantification of the occurrence of gang and group violence in Benin.

### Strengths and limitations

This study has a number of strengths which ensure the reliability of the results. The sample size was representative of in-school adolescents in Benin and large enough to allow for statistical analyses. The questionnaire has been used in several settings with similar characteristics. However the results must be viewed in light of their limitations. Firstly the data were cross-sectional, which does not allow for causal interpretations. Secondly, only in-school adolescents were surveyed which means that the study is silent on the characteristics of those that did not attend school on the day of the survey. Despite being an anonymous survey, social and cultural pressures may have been such that participants responded in a socially desirable manner for some questions. It is therefore likely that the prevalence of suicide reported here is an underestimation. Also a significant number of students did not answer the questions regarding alcohol use, illicit drug use, and bullying. These cases were dropped from the regression analysis and may result in bias in the results. Using multiple imputation suggested that the results for those reporting suicidal ideation may have been biased due to missing data. Bias for the suicide attempts outcomes appear to be less likely. Lastly the measures used in the GSHS do not appear to have been tested for their validity and reliability.

### Conclusions

Suicidal behaviours were found to be associated with a number of risk attributes among in-school adolescents in the Republic of Benin. Several possible avenues for prevention are possible given the structured and “captive audience” nature of school settings. Firstly greater emphasis should be placed on encouraging healthy peer relationships between students. Social cohesiveness and tolerance have been shown to improve the mental well-being of young people. Secondly, encouraging student openness about problems and concerns with designated school counsellors might enable timely intervention. Thirdly, involving parents in problematic school situations which have the potential to have long lasting consequences for the mental health of adolescents is important for increasing accessible levels of support. Fourthly, the prevailing stigma attached to being mentally unwell needs to be faced head on. Just as efforts to reduce disease-related stigma have been given ample attention in SSA settings, the same level of attention should be given to improving the appreciation of mental health as a component of overall health. Ultimately, given the limited data available on adolescent mental health in SSA, particularly where it concerns the well-being of students, is a challenge that must be met with more research. Very little is known about competing pressures that in school adolescents may face such as needing to work outside of the home to provide additional incomes or being caretakers of younger siblings.
